# University of Louisville

**Published:** 1851-06

**Authors:** 


### THE WESTERN JOURNAL.

THIRD SERIES.—VOL. VII.—NO. VI.

LOUISVILLE, JUNE 1, 1851.

### UNIVERSITY OF LOUISVILLE.

Professor Eve has resigned the chair of Surgery in the University of Louisville and it is again filled by Professor Gross. In making this announcement, we scarcely know whether we experience most pleasure or pain. It is pleasant to welcome back an able, well-tried, and faithful colleague; it is painful to part with one who, though known for a shorter time, has been known long enough to have proved himself possessed of all the qualitiees that could be desired in a friend. Dr. Eve and Dr. Gross are both teachers of rare abilities—most acceptable to their pupils, accredited by the profession as among the first surgeons in our country—and with either of them we need hardly say, the school would do well. It is but simple justice to Dr. Eve to state, that he resigned the place in favor of Dr. Gross. He was pleased with the institution, with his colleagues, with his class—pleased with Louisville and Kentucky, and would have remained in the school if Dr. Gross had been content to remain in New York; but on receiving his appointment, last autumn, he expressed a determination not to continue in the chair if Dr. Gross should wish to return to it in the spring, and this resolution he has carried into effect. In doing so he felt that he was making a sacrifice. He had given up his professorship in the Medical College of Georgia and is now without a place, but he conceived that Dr. Gross had higher claims to the chair and he relinquished it cheerfully in his behalf. Such acts of disinterestedness are not exhibited so often that the world is apt to pass by them unobserved, and Dr. Eve is not likely, with all his fine capacities as a teacher and his amiable social qualities, to remain long unemployed.

The return of Dr. Gross to the school will be highly gratifying to that large body of its alumni who have listened to his lectures, and, under the circumstances, must inspire renewed confidence in its stability and permanent success. We should be glad if, for a good space, we might be spared the trouble of recording any other changes in the institution. Few things try the prosperity of a medical school more seriously than ceaseless rotation among its officers. It may be well enough in politics, but science demands something more fixed, stable, and certain.

